# Analysis of risk factors for early-onset ventilator-associated pneumonia in a neurosurgical intensive care unit

**DOI:** 10.1186/s12879-022-07053-7

**Published:** 2022-01-20

**Authors:** Guojie Teng, Ning Wang, Xiuhong Nie, Lin Zhang, Hongjun Liu

**Affiliations:** 1 Department of Pulmonary and Critical Care Medicine, Xuanwu Hospital Capital Medical University, No. 45 Changchun Street, Xicheng District, Beijing, 10053 China; 2 Department of Neurosurgery, Xuanwu Hospital, Capital Medical University, China International Neuroscience Institute (China-INI), No. 45 Changchun Street, Xicheng District, Beijing, 10053 China; 3 Department of Evidence-Based Medicine, Xuanwu Hospital Capital Medical University, Beijing, 100053 China

**Keywords:** Early-onset ventilator-associated pneumonia, Risk factors, Aging, Hypothermia therapy, Neurosurgical intensive care unit

## Abstract

**Background:**

Ventilator-associated pneumonia (VAP) is a severe infection among patients in the neurosurgery intensive care unit (NICU).

**Methods:**

We retrospectively evaluated risk factors for early-onset ventilator-associated pneumonia (EOVAP) from January 2019 to December 2019 at a NICU. A total of 89 NICU patients who were intubated within 48 h of onset and whose mechanical ventilation time was at least 7 days were enrolled. We evaluated EOVAP that occurred within the first 7 days after the onset of mechanical ventilation. The enrolled patients had no history of chronic lung disease and no clinical manifestations of infection before intubation. Clinical data of patients were recorded, and the incidence of and risk factors for EOVAP were analyzed. Patients were also grouped by age (≥ 65 vs. < 65 years) and whether they had received hypothermia treatment or not.

**Results:**

Among 89 mechanically ventilated patients (49 men and 40 women; the mean age ± SD was 60.1 ± 14.3 years), 40 patients (44.9%) developed EOVAP within 7 days and 14 patients (15.7%) had a multidrug resistant bacterial infection. Binary logistic regression analysis indicated that older age (≥ 65 years) (odds ratio [OR]:3.53, 95% confidence interval [CI]:1.27–9.79, P = 0.015) and therapeutic hypothermia (OR:3.68, CI:1.10–12.31, p = 0.034) were independent predictors of EOVAP. Levels of peripheral blood leukocytes, neutrophils and platelets were lower in the therapeutic hypothermia group than those who did not receive hypothermia treatment.

**Conclusions:**

This study found that older age (≥ 65 years) and therapeutic hypothermia were independently associated with the risk of EOVAP in NICU patients.

## Background

Ventilator-associated pneumonia (VAP) remains a common complication among neurosurgery intensive care unit (NICU) patients who require invasive mechanical ventilation. Several measures are available to decrease the incidence of VAP, and these include elevation of the head of the bed, maintenance of tracheal cuff pressure, spontaneous awakening trials and starting enteral nutrition as early as possible [1, 2]. However, despite the application of these interventions, VAP is reported to affect 5–40% of patients receiving mechanical ventilation for more than 2 days[3, 4]. VAP results in markedly prolonged hospital stays[5] and increased ventilator days[6], with attributable mortality estimated to be approximately 13%[7].

Patients with brain injury are highly susceptible to nosocomial pneumonia. Published studies have reported the incidence to range from 22 to 71%[8]. Hence, there is an urgent need to prevent VAP occurrence by early identification of risk factors in patients with brain injury requiring mechanical ventilation. In this context, we aimed to evaluate risk factors for early-onset ventilator-associated pneumonia (EOVAP) in NICU patients undergoing mechanical ventilation for at least 7 days.

## Methods

### Study population

This retrospective observational, single-center cohort study was conducted at Xuanwu Hospital Capital Medical University, China. Neurosurgery is a key specialty in this hospital and it has a total of 38 NICU beds. From January to December 2019, we retrospectively analyzed clinical data of all NICU patients who fulfilled the inclusion criteria. Inclusion and exclusion criteria are reported below:

### Inclusion criteria

(1) Age ≥ 18 years; (2) no fever, cough and sputum, or history of antibiotic use 1 week prior to admission; (3) patients in whom mechanical ventilation was initiated within 48 h of a sudden onset of cerebrovascular disease; and (4) patients with at least 7 days of mechanical ventilation time. We evaluated EOVAP that occurred from the start to the 7th day of mechanical ventilation.

### Exclusion criteria

(1) Pre-existing chronic pulmonary diseases, including pneumonia, lung abscess, chronic bronchitis, chronic obstructive pulmonary disease, bronchial asthma, bronchiectasis, interstitial lung disease and pleural effusion; (2) presence of fever, cough, sputum, and prior antibiotic use; (3) chest radiograph or CT examination before admission showing atelectasis or pneumonia and (4) patients with acute and chronic liver failure, kidney failure, cancer or severe immunodeficiency[9].

### EOVAP definitions

To maintain consistency with the literature, EOVAP was defined as pneumonia occurring within the first 7 days after the onset of mechanical ventilation[10–12].

VAP criteria were as follows: [13, 14] presence of new and/or progressive pulmonary infiltrates on a chest radiograph in a patient ventilated for more than 48 h plus 2 or more of the following:

(1) Temperature > 38 °C; (2) leukocytosis (white blood cell count ≥ 12,000 cells/ mm3) or leukopenia (white blood cell count < 4,000 cells/mm3); and (3) presence of purulent tracheal aspirate.

### Microbiological evaluation

EOVAP was diagnosed by noninvasive sampling and semiquantitative culture as recommended in the guideline literature[15]. All patients who were admitted to the NICU and had been intubated received tracheobronchial aspiration (TBAS) through a closed-suction system. The TBAS was sent to the hospital's microbiology laboratories for the detection of bacteria and fungi. In the microbiology laboratory, the TBAS was plated on agar medium (3 days of culture for aerobic bacteria and 2 weeks for fungi) using a semiquantitative culture method. Bacterial identification and antibiotic susceptibility tests using standard methods were performed for samples that showed positive growth, as recommended in CDC guidelines[16]. Within 24 h after tracheal intubation, all patients received tracheobronchial aspiration culture and repeated aspiration culture every 48–72 h after that.

Multidrug-resistant (MDR) was defined as nonsusceptibility to 1 or more agents in 3 or more antimicrobial classes (ie, aminoglycosides, third-/fourth-generation cephalosporins, fluoroquinolones, beta-lactam/beta-lactamase inhibitors, and carbapenems) for Gram-negative organisms; nonsusceptibility to oxacillin and/or cefoxitin (anti-staphylococcal beta-lactams) for Gram-positive *Staphylococcus aureus*; and non-susceptibility to vancomycin and/or teicoplanin (glycopeptides) for Gram-positive *Enterococcus spp*.[17, 18].

### Data collection

The following data were obtained: (1) Patient's information: age (categorized as ≥ 65 and < 65 years), sex, smoking, body mass index (BMI), pre-existing comorbidities (coronary artery disease, hypertension, diabetes), intubation time (hospital admission or prehospital intubation), Acute Physiology and Chronic Health Evaluation II (APACHE II) and Glasgow Coma Scale/Score(GCS); (2) laboratory data: albumin (ALB) C-reactive protein (CRP), procalcitonin (PCT), alanine aminotransferase (ALT), serum creatinine (Scr), full blood count, sputum culture and chest X-ray; (3) medications administered: norepinephrine, glucocorticoids, mannitol, therapeutic hypothermia, antibiotics administered, and sedative and analgesics after the intubation; and (4) outcomes of EOVAP and non-EOVAP groups: mechanical ventilation time, ICU stay, hospital stay, and 28-day mortality.

### Statistical analysis

All statistical analyses were performed using SPSS statistical software version 19.0. Continuous data were presented as mean ± standard deviation for normally distributed variables and median (interquartile range, IQR; 25th–75th percentile) for those not normally distributed. Independent-Samples T-Test or Mann–Whitney non-parametric test was used to compare differences in continuous variables depending on their distribution. Chi-square test or Fisher’s Exact test was used to compare categorical data. Binary multivariable logistic regression analysis was performed for parameters with p < 0.10 on univariate analysis and the odds ratios (ORs) with 95% confidence intervals (95%CIs) were calculated. All tests were two-tailed, with the significance level set at p < 0.05.

## Results

### Patient characteristics

**Fig. 1:**
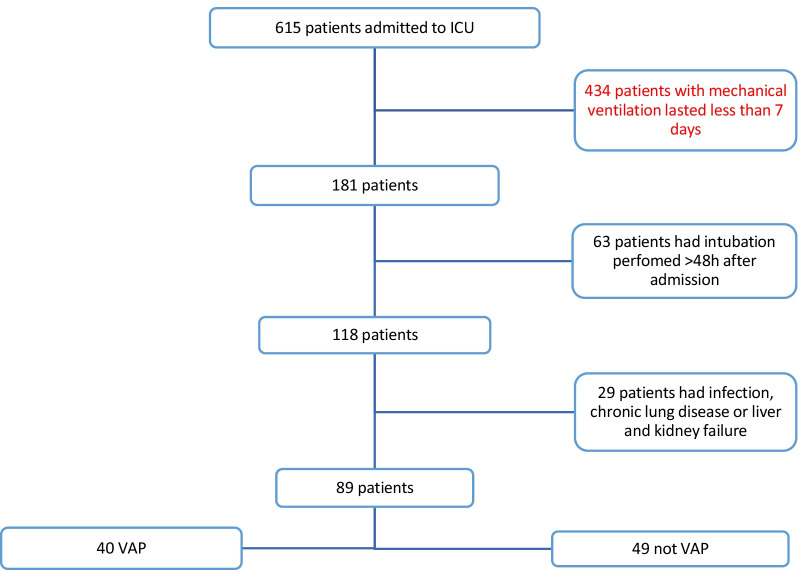
Flow chart of patient selection criteria

During a 12-month period, 615 mechanically ventilated patients were admitted to the NICU at our hospital. After an initial medical record review, the following records were removed from the analysis: 434 records for either extubation or death within 7 days; 63 records for intubation performed more than 48 h after admission; and 29 records for infection, chronic lung disease, or liver and kidney failure before intubation. A total of 89 patients who underwent mechanical ventilation satisfied the inclusion criteria. Among 89 mechanically ventilated patients (49 men and 40 women; the mean age ± SD was 60.1 ± 14.3 years), APACHE II was 13.8 ± 3.8 and the GCS was 7.8 ± 2.2. Of the 89 patients, there were 52 cases of subarachnoid hemorrhage, 29 cases of intracerebral hemorrhage, and 8 cases of massive cerebral infarction. Of the 89 patients, 40 patients (44.9%) developed EOVAP within 7 days (Fig. 1).

### Antibiotic use and multidrug-resistant bacteria (MDRB)

All 89 patients were treated with prophylactic antibiotics (including 33 cases with cefminox, 32 cases with piperacillin tazobactam and 24 cases with ceftriaxone) within 24 h after intubation. Antibiotics were continuously administered and adjusted according to the results of the bacterial culture. Within 7 days of intubation, 12 patients (13.5%) received carbapenem treatment for more than 3 days, of which 3 patients had their medication adjusted based on the results of sputum culture, and 9 received empirical antibiotic escalation treatment. The sputum culture results within 7 days in 14 patients (15.7%) showed MDRB (3 cases of *Staphylococcus aureus*, 3 cases of *Burkholderia cepacia*, 3 cases of *Klebsiella pneumoniae*, 3 cases of *Corynebacterium striatum*, 1 case of Acinetobacter pitti, 1 case of *Acinetobacter beijerinckii*, and 1 case of *Acinetobacter baumannii*). However, statistical results showed no significant correlation between the administration of carbapenem antibiotics and the appearance of Gram-negative MDRB in the short term.

### Risk factors for EOVAP

All 89 patients had no infection or underlying lung diseases on presentation. They were intubated within 48 h of the onset of cerebrovascular disease and mechanical ventilation was maintained for more than 7 days. All patients were placed in a position with the head of the bed raised > 30° on the day of intubation which lasted for 7 days. Within 2 days after intubation, all patients were provided with gastrointestinal nutritional support, acid inhibitors (H2 blocker or proton pump inhibitor), and blood glucose monitoring. Of the 89 patients, 2 underwent tracheotomy on the 5^th^ and 6^th^ days after intubation. The univariate analysis of the EOVAP group and the non-EOVAP group showed that: ≥ 65 years old, PCT1(PCT at the time of diagnosis of pneumonia), CRP1(CRP at the time of diagnosis of pneumonia), and Platelet1(Platelet at the time of diagnosis of pneumonia) were statistically different between the groups. There were no significant differences between the two groups in the use of glucocorticoids, MDRB, and 28-day mortality, but the total mechanical time of the EOVAP group was longer than that of the non-EOVAP group (Table 1).

**Table 1:** Characteristics of NICU patients comparing EOVAP vs. non-EOVAP groups

Variables	EOVAP (n = 40)	Non-EOVAP (n = 49)	*P-*value
Patient's information			
Ageing(≥ 65y)	23	17	0.031
Male gender	23	26	0.675
Smoking	3	6	0.506
BMI (body mass index)	25.4 ± 6.0	25.1 ± 3.9	0.845
Coronary artery	5	5	0.997
Diabetes	5	11	0.224
Hypertension	24	26	0.512
Prehospital intubation	2	5	0.287
APACHE II	14.3 ± 4.0	13.4 ± 3.7	0.274
GCS	7.8 ± 2.0	7.9 ± 2.4	0.864
Drug use (cumulative use time > = 3d)			
Norepinephrine	19	20	0.527
Glucocorticoid	11	13	0.918
Mannitol	34	43	0.705
Therapeutic hypothermia	13	8	0.074
Carbapenem antibiotics	5	7	0.806
Propofol or propofol medium and long chain fat emulsion	26	36	0.387
Midazolam	27	36	0.641
Remifentanil or fentanyl	36	44	1.000
Proton pump inhibitor	34	46	0.289
H 2 receptor antagonist	6	3	0.291
Laboratory testing (At the time of intubation)			
ALB (g/l)	33.0 ± 6.7	32.5 ± 6.7	0.752
CRP (mg/l)	61.4 ± 46.7	60.6 ± 47.3	0.936
PCT (ng/mL), median (IQR)	0.220 (0.10–0.5200)	0.120 (0.080–0.500)	0.148
ALT (U/l), median (IQR)	29.00 (18.25–33.00)	30.00 (19.00–33.5)	0.944
Scr (mmol/l), median (IQR)	65.50 (51.25–83.00)	58.00 (43.00–81.00)	0.180
White blood cell count (× 10^9^/l)	13.4 ± 5.0	12.5 ± 3.8	0.317
Neutrophils (× 10^9^/l)	11.9 ± 4.7	12.6 ± 12.2	0.734
Lymphocyte (× 10^9^/l)	0.95 ± 0.46	0.87 ± 0.38	0.373
Platelet (× 10^9^/l)	180.9 ± 66.5	175.3 ± 61.7	0.683
Laboratory testing (on day 7 after intubation)			
ALB (g/l)	33.8 ± 5.1	32.8 ± 5.1	0.367
ALT (U/l), median (IQR)	36.50 (30.00–62.75)	46.00 (31.00–60.5)	0.468
Scr (mmol/l), median (IQR)	63.5 (45.50–93.00)	52.00 (42.00–76.5)	0.198
Laboratory testing (EOVAP group: when pneumonia was diagnosed, non-EOVAP group: on day 7 after intubation)			
CRP (mg/l), median (IQR)	76.45 (53.38–111.00)	55.00 (30.25–64.75)	0.008
PCT (ng/ml), median (IQR)	0.22 (0.13–0.44)	0.120 (0.075–0.310)	0.047
White blood cell count (× 10^9^/l)	9.4 ± 3.6	9.7 ± 3.2	0.689
Neutrophils (× 10^9^/l)	7.8 ± 3.4	7.9 ± 3.0	0.891
Lymph (× 10^9^/l) median (IQR)	0.8(0.58–1.22)	0.97 (0.73–1.36)	0.108
Platelet (× 10^9^/l)	147.4 ± 63.4	177.6 ± 76.2	0.044
MDRB	7	7	0.679
Outcomes of EOVAP and non-EOVAP groups			
Mechanical ventilation time (h) median (IQR)	384(301–493)	290(207–432)	0.02
ICU stays (d)	19.4 ± 9.6	20.3 ± 11.0	0.676
Hospital stays (d)	24.8 ± 14.6	23.5 ± 11.7	0.628
28-day mortality (d)	8	7	0.474

Five factors with P < 0.1 in the univariate analysis: ≥ 65 years, therapeutic hypothermia, PCT1, CRP1, and Platelet1 were included in the binary multivariable logistic regression analysis. Logistic regression analyses showed that older age (≥ 65 years) ((OR: 3.53, CI: 1.27–9.79–0.738, p = 0.015) and therapeutic hypothermia (OR:3.68, CI:1.1–12.31, p = 0.034) were independent predictors of EOVAP (Table 2).

**Table 2:** Results of binary multivariable logistic regression analysis showing risk factors for EOVAP in NICU patients

Variable	β	SE	Wald value	EXP(B) (95%CI)	P-value
Therapeutic hypothermia	1.303	0.616	4.476	3.68 (1.10–12.31)	0.034
Older age (≥ 65 years)	1.262	0.520	5.884	3.53 (1.27–9.79)	0.015
CRP1	0.006	0.004	1.870	1.01 (1.00–1.01)	0.171
PCT1	0.004	0.216	0.000	1.04 (0.66–1.53)	0.984
Platelet1	−0.002	0.004	0.367	0.998 (0.99–1.01)	0.545

### Older age (≥ 65 years) and therapeutic hypothermia

Patients were grouped by age (≥ 65 vs. < 65 years) and whether they had received hypothermia treatment or not, and differences in clinical characteristics between these groups were compared. Underlying diseases, inflammatory indicators, drug use and mortality, were not significantly different between the two age groups. However, levels of peripheral blood leukocytes, neutrophils and platelets were lower in the therapeutic hypothermia group than the group that did not receive hypothermia treatment, with no significant differences in other indicators, including measures of inflammation and sedative use (Table 3).

**Table 3:** Characteristics of NICU patients comparing hypothermia therapy vs. non-hypothermia therapy groups

Variables	Hypothermia therapy (n = 21)	Non-hypothermia therapy (n = 68)	*P*-value
Laboratory testing (7 days after intubation)			
CRP (mg/l), median (IQR)	57.53 (22.10–79.20)	58.76 (30.57–92.35)	0.565
PCT (ng/ml), median (IQR)	0.13 (0.10–0.21)	0.17 (0.08–0.53)	0.431
ALT (U/l), median (IQR)	35.43 (33.13–39.61)	44.50 (30.00–61.75)	0.889
Scr (mmol/l), median (IQR)	50.00 (38.00–76.00)	58.50 (43.50–84.00)	0.214
White blood cell count (× 10^9^/l)	7.60 ± 2.31	10.10 ± 3.40	0.002
Neutrophils (× 10^9^/l)	6.22 ± 2.19	8.34 ± 3.04	0.004
Lymph (× 10^9^/l)	0.86 ± 0.48	1.13 ± 0.61	0.072
Platelet (× 10^9^/l) *	137.52 ± 63.20	180.60 ± 79.59	0.026
Use of sedative and analgesics (cumulative use time > = 3d)			
Propofol or propofol medium and long chain fat emulsion	13 (61.9%)	49 (72.1%)	0.376
Midazolam	17 (81.0%)	46 (67.6%)	0.258
Remifentanil or fentanyl	18 (85.7%)	62 (91.2%)	0.755

*The platelet count (7 days after intubation): mortality group (within 28 days) vs. alive group *P* = 0.006

## Discussion

In our retrospective analysis of risk factors for EOVAP in NICU patients, the results showed that older age (≥ 65 years) and therapeutic hypothermia were independent predictors of EOVAP. The levels of peripheral blood leukocytes, neutrophils, and platelets in hypothermia-treated patients were lower than those of non-hypothermia-treated patients. The platelet count in patients with therapeutic hypothermia was associated with 28-day mortality.

Even though most MDRB are isolated from patients with late-onset VAP, accumulating evidence shows that drug resistance is also a problem in patients who develop early-onset VAP[19, 20]. In this study, NICU patients with no history of antibiotic use before admission had a 15.7% (14/89) incidence of MDRB during 7-day mechanical ventilation. Several studies have shown that the percentage of MDR pathogens among patients with early-onset VAP varied from as low as 10% to as high as 51%[21]. In our study, the incidence of MDRB was only 15%, which may be due to strict patient selection criteria. We excluded patients with previous underlying lung disease and liver and kidney dysfunction, as these patients are high-risk groups for MDRB. There was no correlation between the administration of carbapenem antibiotics in the short term and the emergence of Gram-negative MDRB. It is speculated that MDRB may originate from exogenous sources such as contaminated respiratory instruments, infected aerosols from the ICU environment and contaminated hands and apparel of healthcare workers[22, 23].

Although univariate analysis showed statistical differences in CRP and PCT between the EOVAP group and the non-EOVAP group, binary logistic regression indicated that CRP and PCT were not independent predictors of EOVAP, a finding consistent with other studies [29–32]. In our study, older age (≥ 65 years) and hypothermia therapy were independent predictors of EOVAP. A European multicenter cohort study reported that VAP was not increased among the elderly, but the associated mortality in these patients was higher[24]. This is contrary to our findings. Our evaluation of 89 patients with no history of chronic lung disease, no infection before intubation, and a similar duration of mechanical ventilation after intubation, showed that age ≥ 65 was an independent predictor of EOVAP. The divergent findings between the two studies may be attributed to the stringent patient inclusion criteria adopted in our study. The high incidence of EOVAP in the elderly (≥ 65 years) may be due to the gradual decline in respiratory function with age, the gradual atrophy of respiratory muscles, the decline in lung elasticity and the decline in the ability to expel sputum. At the same time, the respiratory mucosa of the elderly shrinks, the mucosal function decreases, and the local defense function of the respiratory tract decreases, leading to an increase in the incidence of VAP.

Several published reports have indicated that hypothermia therapy is one of the most important risk factors for early-onset pneumonia. Esnault reported that the incidence of EOVAP after severe traumatic brain injury was more than 61% and that hypothermia was one of the major risk factors for EOVAP[12]. Sébastien reported that after out-of-hospital cardiac arrest, therapeutic hypothermia was associated with an increased risk of early-onset pneumonia[25]. Our findings further corroborate these observations. Hypothermia impairs immune functions by inhibiting the secretion of proinflammatory cytokines and suppressing leukocyte migration and phagocytosis [26]. Hypothermia-induced insulin resistance and hyperglycemia may further increase infection risk[25], leading to an increase in the incidence of VAP.

Bro-Jeppesen and Dufner reported that hypothermia treatment could lead to a decrease in the number of white blood cells and neutrophils[27, 28], which is consistent with the results of our study. However, the relationship between hypothermia and platelet count is controversial. Nielsen reported that thrombocytopenia occurred after therapeutic hypothermia in patients with cardiac arrest[29, 30]. But Takashi reported that therapeutic hypothermia had no significant effect on platelet counts in patients with severe traumatic brain injury. Our study found that after 7 days of mechanical ventilation, the platelet count of patients treated with hypothermia was significantly reduced, and it was associated with 28-day mortality (P = 0.006). The cause of thrombocytopenia due to hypothermia is still unclear. Some studies have shown that hypothermia enhances shear-induced platelet aggregation[31] and decreases platelet function[32]. These results suggest that the monitoring of platelets in patients with hypothermia is essential. Further evaluation is needed when clinicians detect thrombocytopenia in patients treated with hypothermia.

The limitations of this study deserve consideration. They include the single-center retrospective design and the small sample size; a prospective design with a larger sample would yield more robust results. Our study used the VAP definition for analysis, but the use of ventilator-related events may have better clinical applicability.

## Conclusion

We retrospectively analyzed the clinical data of 89 NICU patients who received mechanical ventilation between January and December 2019. All 89 patients had no infection or underlying lung diseases. Our findings showed that older age (≥ 65 years) and therapeutic hypothermia were independently associated with the risk of EOVAP in NICU patients.

## Data Availability

The datasets used and/or analyzed during the current study are available from the corresponding author on reasonable request.

## Abbreviations

VAP: Ventilator-associated pneumonia
NICU: Neurosurgery intensive care unit
EOVAP: Early-onset ventilator-associated pneumonia
TBAS: Tracheobronchial aspiration
BMI: Body mass index
APACHE II: Acute Physiology and Chronic Health Evaluation,
GCS: Glasgow Coma Scale/Score
MDRB: Multidrug-resistant bacteria
ALB: albumin
CRP: C-reactive protein
PCT: Procalcitonin
ALT: Alanine aminotransferase
Scr: Serum creatinine
